# Modelling of psychosocial and lifestyle predictors of peripartum depressive symptoms associated with distinct risk trajectories: a prospective cohort study

**DOI:** 10.1038/s41598-018-30874-z

**Published:** 2018-08-24

**Authors:** Sarah English, Amber Steele, Alison Williams, Jayne Blacklay, Olanrewaju Sorinola, Lorenz Wernisch, Dimitris K. Grammatopoulos

**Affiliations:** 1 Translational Medicine, Warwick Medical School, Coventry, UK; 2 MRC Biostatistics Unit, Institute of Public Health, Cambridge, UK; 3 South Warwickshire Hospital Foundation Trust, Warwick, UK; 4 Institute of Precision Diagnostics and Translational Medicine, Department of Pathology, University Hospital Coventry and Warwickshire, Coventry, UK

## Abstract

Perinatal depression involves interplay between individual chronic and acute disease burdens, biological and psychosocial environmental and behavioural factors. Here we explored the predictive potential of specific psycho-socio-demographic characteristics for antenatal and postpartum depression symptoms and contribution to severity scores on the Edinburgh Postnatal Depression Scale (EPDS) screening tool. We determined depression risk trajectories in 480 women that prospectively completed the EPDS during pregnancy (TP1) and postpartum (TP2). Multinomial logistic and penalised linear regression investigated covariates associated with increased antenatal and postpartum EPDS scores contributing to the average or the difference of paired scores across time points. History of anxiety was identified as the strongest contribution to antenatal EPDS scores followed by the social status, whereas a history of depression, postpartum depression (PPD) and family history of PPD exhibited the strongest association with postpartum EPDS. These covariates were the strongest differentiating factors that increased the spread between antenatal and postpartum EPDS scores. Available covariates appeared better suited to predict EPDS scores antenatally than postpartum. As women move from the antenatal to the postpartum period, socio-demographic and lifestyle risk factors appear to play a smaller role in risk, and a personal and family history of depression and PPD become increasingly important.

## Introduction

Depression during pregnancy, around childbirth or within the first year postpartum, collectively termed as perinatal depression (PND), is now recognised as a major health burden both for the mother, her family and the offspring^1,2^. Across most ethnic groups perinatal depression is experienced by 10–20% of women^3,4^ exhibiting similar prevalence to depression in the general population. It is now considered one of the most common non-obstetric complications associated with childbearing.

Similar to general depression, PND is a heterogeneous complex disorder that involves bidirectional interplay of stress vulnerability, depression and health outcomes^5^. Individual chronic/acute burdens associated with previous history of illness that precipitate or exacerbate depressive symptoms play a major role in stress vulnerability as well as factors relevant to biological and psychosocial environment such as demographics and socioeconomic status, social support and lifestyle^6,7^. Major meta-analysis studies identified a number of key predictors of postpartum depression (PPD) including history of depression, antenatal depression and anxiety, major stressful life events, social support levels, low self-esteem and income and negative cognitive attributional style^3,8,9^. History of depression, antenatal anxiety, low self-esteem, social support, income and educational attainment are also predisposing factors for antenatal depression (AND)^10–13^.

Routine screening is strongly encouraged by the American College of Obstetricians and Gynecologists (ACOG) Committee on Obstetric Practice in the US, US Preventive Services Task Force and the National Collaborating Centre for Mental Health in the UK^14–16^. The Edinburgh Postnatal Depression Scale (EPDS) is the most commonly used standardized screening tool for both antenatal (AND) and postpartum (PPD) depressive symptoms and is recommended by the National Institute of Clinical Excellence^16^. Although there is no consensus agreement on the most appropriate cut-off, for screening purposes a cut-off score of 10 is widely cited to indicate possible major or minor depressive disorder^17,18^, whereas the cutoff of 13 is typically used for identifying major depressive disorder (MDD)^15^.

Studies sampling EPDS scores at different time points during pregnancy and postpartum report considerable variability in the pattern of symptom onset, recurrence and duration, and severity^19^; for example although most studies agree that antenatal depressive symptoms are generally associated with PPD, not all women with PPD report raised EPDS during pregnancy. In fact, studies including our own pilot^19–22^ suggest that women exhibit diverse, either ascending or descending trajectories in EPDS scores during pregnancy and postpartum. Whether specific risk factors determine or influence the direction of EPDS score trajectories is poorly understood, and very few studies have addressed this. Thus, identifying key psychosocial, lifestyle and pregnancy covariates as predictors of changes between antenatal and postpartum EPDS scores might be important and improve EPDS predictive accuracy and potential use in a screening protocol.

By recording antenatal and postpartum EPDS scores together with profiling psychosocial, lifestyle and pregnancy related information, routinely gathered during antenatal hospital visits, in this prospective study we initially investigated whether distinct patterns of onset and persistence of PND symptoms from pregnancy to the postpartum period are associated with different risk profiles. This analysis was based on modeling by discretization applying well-established cutoffs. In addition, data analysis using a full undiscretized EPDS scale, explored the usefulness of covariates as predictors of either antenatal or postpartum EPDS scores in order to identify covariates that can support a “predictive” model capable of forecasting raised EPDS scores. A second aim of the analysis was to detect whether systematic differences between antenatal and postpartum EPDS scores exist, potentially hinting at different aetiologies.

## Results

### Antenatal-postpartum EPDS score distributions and predictive performance

The complete data set consisted of 480 matched pairs with completed antenatal and postpartum EPDS scores. Distribution and frequencies of EPDS scores are displayed in Suppl. Fig. 1a [studies comparing the antenatal (T1) and postpartum (T2) EPDS scores are also shown in the *Penalised Regression analysis and prediction* section]. A detailed description of the cohort EPDS score characteristics is presented in the Supplementary Results. Using the cut-off of 10 to categorize EPDS scores as ‘high’ or ‘low’ risk, the risk trajectory defined by EPDS scores during pregnancy and postpartum period determined classification of participants into four groups for the initial analysis (Table 1). Group 0 represents a low risk trajectory, Group 3 an overall high-risk trajectory, and Groups 1 & 2 transient risk trajectories with either an increase (group 1) or decrease (group 2) in postpartum EPDS scores.

**Table 1:** Distribution of the cohort within created Group variables.

Group	Total N	%
0 - Overall Low Depressive Risk	379	79.0
1 - Low antenatal risk, High risk postpartum	46	9.6
2 - High antenatal risk, Low risk postpartum	30	6.3
3 - Overall High Depressive Risk	25	5.2

**Figure 1:**
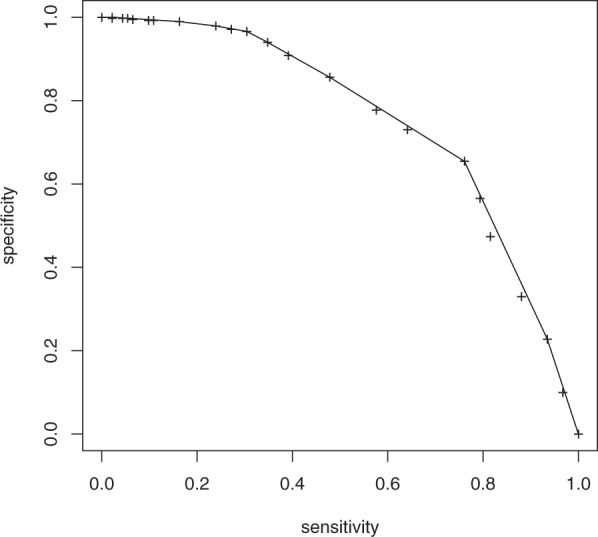
ROC curve (convex hull) for prediction of postpartum EPDS score ≥10 from antenatal EPDS scores (AROC 0.756) at various cut-offs (crosses).

Previous studies also investigated predictive accuracy of the antenatal EPDS for postpartum EPDS at different cut-offs^21^. For a comparison with these studies we established how far an antenatal EPDS score ≥10 during the 3^rd^ trimester could directly predict an EPDS score ≥10 6–10 w postpartum (Table 2). To assess the predictive power under a range of all possible cut-offs we measured the area under the ROC curve obtaining a value of 0.76 shown in Fig. 1, which confirms previous studies^21^. Further details on predictive performance of the antenatal and postpartum EPDS score are shown in Table 2. These results are essentially identical to previous studies^21^ albeit requiring a slight shift in the EPDS cut-off to obtain similar levels of NPV and PPV.

**Table 2:** Predictive performance of antenatal EPDS score at various cut-offs for a postpartum EPDS score ≥10 (standard error in brackets).

	Cut-off ≥15	Cut-off ≥10	Cut-off ≥5
Sensitivity % (95% CI)	16.3 (3.9)	39.1 (5.1)	79.3 (4.2)
Specificity % (95% CI)	99.0 (0.5)	90.8 (1.5)	56.5 (2.5)
PPV % (95% CI)	78.9 (9.4)	50.7 (5.9)	30.5 (3.0)
NPV % (95% CI)	83.1 (1.8)	86.1 (1.7)	91.9 (1.8)

The area under the ROC curve for all cut-offs (see Fig. 3) is 0.756.

### Multinomial logistic regression – group analysis

To investigate whether distinct patterns of onset and persistence of PND symptoms from pregnancy to the postpartum period are associated with different risk profiles, we dichotomised EPDS scores using standard cut-offs to define a binary outcome of depressive risk status. This approach is relevant to clinical guidelines and routine healthcare practice of risk assessment and provides a means of elucidating the heterogeneity of symptoms severity across women. In this way we obtained estimates of effects and their significance that are more robust to potential misspecifications of the regression models. Initial bivariate chi-squared analysis identified 11 potential correlates associated with antenatal/postpartum depressive symptoms, which were further analysed. Complete results are shown in Suppl. Tables 3 and 4. All risk factors with a p < 0.05, either pre- or post-delivery, were included in a multinomial regression model for the group analysis. The overall p-value for this model was 0.0002. The low risk (EPDS <10) group was used as a baseline category. Relative risk values (RR) were calculated to give a better understanding of the magnitude of association between covariates as risk factors and the 3 groups with distinct risk trajectories (Table 3).

**Table 3:** Relative Risk (RR) values for significant variables within each at risk group.

Group	Risk factors
1 – High postpartum risk	Past history of PPD (p = 0.017, RR = 3.78) Past history of depression (p = 0.034, RR = 2.76)
2 – High antenatal risk	Past history of anxiety (p = 0.013, RR = 3.55) Ethnicity - minority (p = 0.006, RR = 5.19) Age - <24 (p = 0.010, RR = 6.39)
3 – Overall high risk	Past history of depression (p = 0.045, RR = 3.51)

For group 1 (T2 EPDS ≥10 only), significant risk factors were found to be ‘Past history of PPD’ and ‘Past history of depression’. In group 2 (T1 EPDS ≥10 only), risk factors were a ‘Past history of anxiety’, Age (<25)’ and ‘Ethnicity (minority)’, although it is important to note that 84.6% of the cohort were White British. An age of 40+ also increased the risk of being in Group 2 (RR = 4.17) but this association was not quite significant and was relevant to <10% of the cohort. In group 3 (both T1 and T2 EPDS ≥10), ‘Past history of depression’ was the only significant risk factor.

### Penalised Regression analysis and prediction

**Figure 2:**
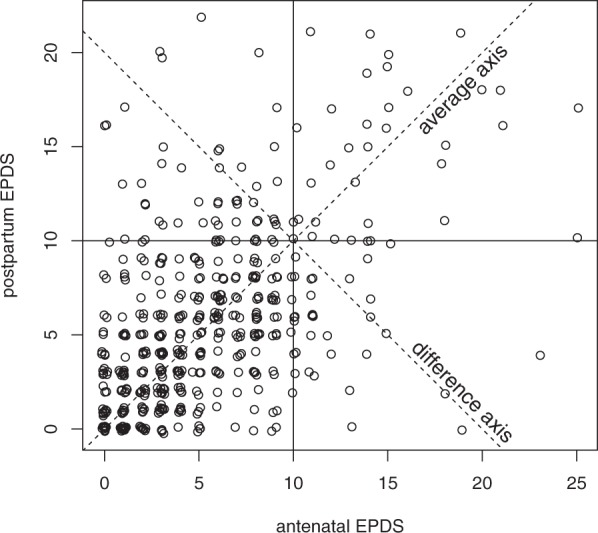
Antenatal vs postpartum EPDS scores for 474 individuals (correlation 0.5002). Points are jittered randomly for better visibility. Sections of the graph represent groups created in the previous analysis.

In the previous section we identified covariates which constitute major risk factors for either group 1 (history of PPD, history of depression), group 2 (history of anxiety, ethnicity, age), or group 3 (history of depression). It is likely that dichotomising EPDS scores results in loss of statistical power; it also depends on specific cut-off values. We therefore repeated the regression analysis on the full range of EPDS scores without dichotomisation. The relationship between the antenatal (T1) and postpartum (T2) EPDS scores, with a correlation of 0.5002, is shown in Fig. 2. The deviance from perfect correlation and alignment can be attributed to any systematic change (drop or increase) in depressive symptoms from before to after delivery, but also to unexplained variability (noise) in the acquisition of EPDS scores.

In the following section we present linear as well as nonlinear regression analyses for the antenatal and postpartum EPDS scores, for the difference between them and finally for their average. The purpose of these four analyses is to explore whether covariates contributing to a high *antenatal* score are different from covariates contributing to a high *postpartum* score (compare also with Fig. 1) and which covariates contribute to both. Details of the regression analysis can be found in the methods section and the supplementary material.

### Antenatal EPDS score

Variables contributing to a linear model for antenatal EPDS scores were selected using penalised regression. Once variables were selected, regression were calculated by standard linear regression. Coefficients with a *p* < 0.05 are shown in Table 4 (the full model is listed in Suppl. Table 5).

**Table 4:** Significant covariates of a linear model for antenatal EPDS scores.

Covariate	Coefficient	p-value
Social status - Routine/semi-routine/HW	1.768	<0.001
Social status - Unemployed/student	3.887	0.004
Education >18 years	−1.373	0.033
BMI ≥30 - Yes	−0.581	0.037
Alcohol pre-pregnancy - Yes	0.813	0.049
Family history of PPD, 1st degree relative - Yes	1.391	0.012
Past history of anxiety - Yes	2.314	<0.001

Positive or negative coefficients indicate by how much the EDPS score increases or decreases with the covariate. The full model is provided in Suppl. Table 5.

Covariates that appeared particularly important in the prediction of antenatal EPDS scores were ‘*past history of anxiety’* contributing around 2 points toward the EPDS score, the most significant contribution in terms of a *p*-value, with further contributions identified from *‘social status as Unemployed/student’*, contributing almost 4 points towards the antenatal EPDS score, and ‘*Semi-routine/routine/HW work*’ contributing about 1.8 points. Other significant contributions come from *a ‘family history of PPD’* as well as ‘*alcohol consumption*’. Marginal protective effects were identified from ‘*education beyond* 18 *years*’ and a higher BMI. Although not significant, further variables contributed to increased prediction accuracy as listed in Suppl. Table 3.

We also fitted a model adding all pairwise interactions. However, the cross validated mean squared prediction error was unchanged indicating that adding interactions does not improve predictive power and we therefore did not further consider such models or any models with higher orders of interaction.

### Postpartum EPDS scores

As above a linear model for postpartum EPDS scores was selected using penalised regression. Coefficients with a *p* < 0.05 are shown in Table 5 (for a full list see Suppl. Table 6). Fewer covariates identified as significant compared to the antenatal EPDS score model. *‘Past history of depression*’, ‘*Past history* as well as *a family history of PPD*,’ contributed about 2.8, 2.2, and 1.8 points to the postpartum EPDS, respectively. Information about pre-existing illness and associated medication as well as level of education improved prediction accuracy although their effect was not statistically significant.

**Table 5:** Significant covariates of a linear model for postpartum EPDS scores.

Covariate	Coefficient	p-value
Past history of PPD - Yes	2.204	0.014
Past history of depression - Yes	2.788	<0.001
Family history of PPD - 1st degree relative - Yes	1.779	0.003

The full model is provided in Suppl. Table 6.

### Differences and commonalities between antenatal and postpartum EPDS scores

**Figure 3:**
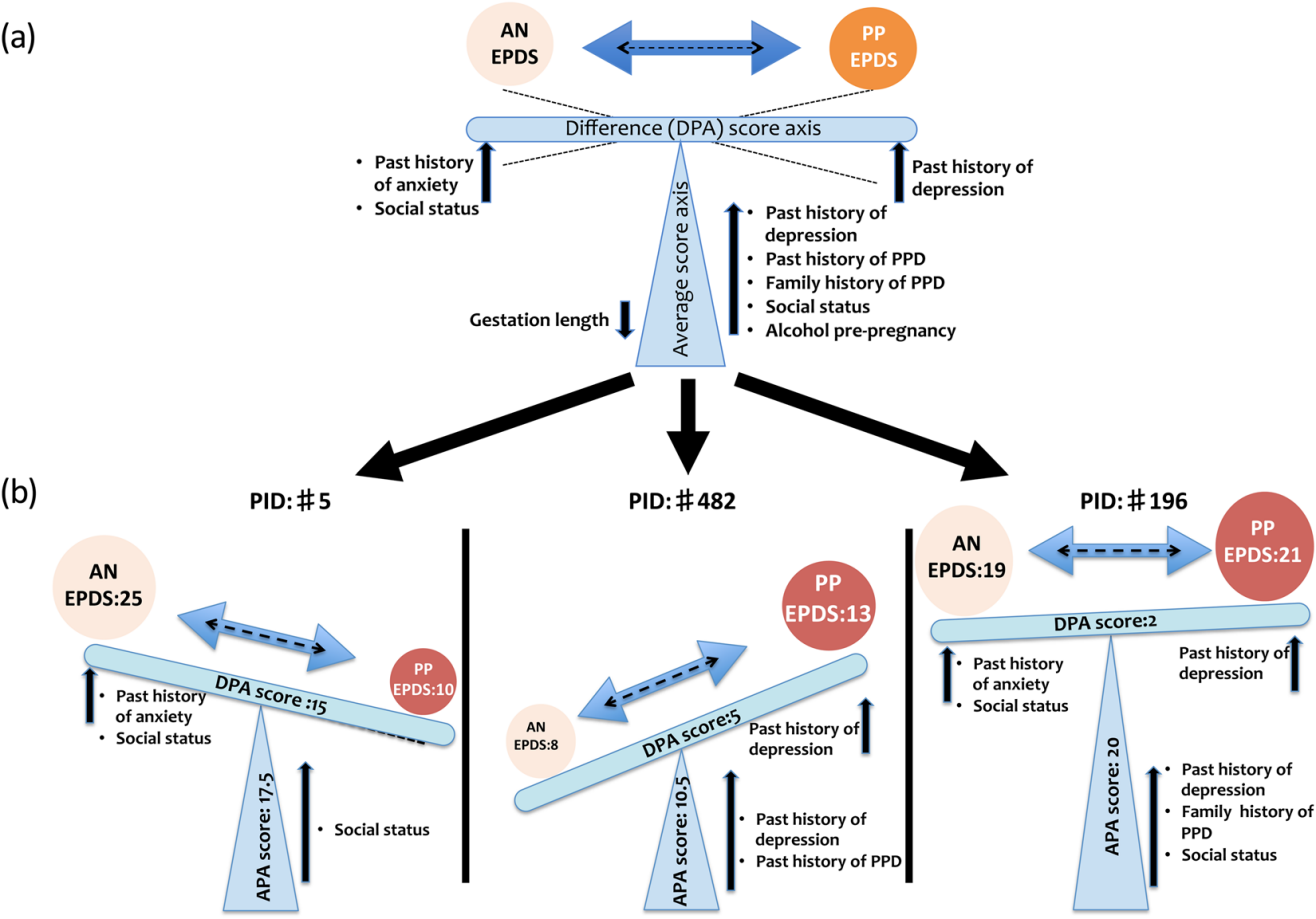
(**a**) A schematic of the relationship between contribution of covariates identified by a linear model to the differences and averages of postpartum and antenatal EPDS scores. The ‘average’ EDPS score at T1 and T2 time points (APA score) can be seen as an indication of overall perinatal depression not specific to either an antenatal or postpartum time point. The ‘difference’ in EPDS scores (DPA score) corresponds to shifts in EPDS score from the antenatal to the postpartum time point indicative of improvement or worsening. (**b**) Representative case studies of cohort patients with covariates that shift DPA and APA scores. Subject #5 who had two covariates with negative coefficients, a ‘previous history of anxiety’ and a ‘social status of student/unemployed’, exhibited a significant drop in the DPA score. Subject #482 had a covariate with positive coefficient, a ‘previous history of depression’ and exhibited an increase in the DPA score. In this patient, the covariates ‘past history of PPD’ as well as ‘past history of depression’ might contribute to the APA score. Subject #196 had covariates with both negative and positive coefficients, a previous history of anxiety’ and ‘previous history of depression’ and exhibited a small increase in the DPA score. In this patient, the APA score was also influenced by at least 3 covariates with positive coefficients, such as ‘family history of PPD’, ‘past history of depression’ and ‘social status’.

We explored systematic differences between antenatal and postpartum EPDS scores in terms of potentially different sets of covariates, and covariates that would explain the drop or rise in antenatal to postpartum EPDS scores observed in many subjects. Therefore, we investigated within-person patterns of change by analysing the contribution of covariates to the differences and averages of postpartum (T2) and antenatal (T1) EPDS scores. The ‘average’ EDPS score [APA score = (T1 + T2)/2] can be seen as an indication of overall perinatal depression not specific to either an antenatal or postpartum time point; identified covariates contributing to the average score would indicate common underlying factors. The ‘difference’ in EPDS scores [DPA score = (T1 − T2)] corresponds to the improvement or worsening in EPDS score from the antenatal to the postpartum time point; covariates contributing to the difference of T1 to T2 score, would indicate differentiating factors. The relationship between difference and average score is illustrated in Fig. 3. The two components are also indicated as average and difference axes in Fig. 2. Their correlation with different sets of predictors is discussed below.

#### Difference between postpartum and antenatal EPDS scores

We analysed covariates, which contribute to the DPA score in order to find correlates explaining why some individuals exhibited a drop in score from before to after delivery while others showed the opposite effect. Table 6 shows significant coefficients of a linear model selected by penalised regression (the full model is shown in Suppl. Table 7). Negative coefficients indicate a decrease from antenatal to postpartum EPDS score, while positive coefficients indicate an increase. The clinical significance of a 1 point change in EPDS score represents a “small change”, 2–3 points a “medium change” and 4 points or higher a “large change”^23^.

**Table 6:** Significant covariates of a linear model for the difference of antenatal to postpartum (DPA) EPDS scores.

Covariate	Coefficient	p-value
Social status - unemployed/student	−2.664	0.040
Past history depression - yes	2.036	0.003
Past history of anxiety - yes	−2.565	<0.001

The full model is provided in Suppl. Table 7.

Based on our regression analysis one would expect covariates that contribute to T1 EPDS score (Table 4) but not T2 EPDS score (Table 5) to exert negative coefficients in this analysis, since they can explain antenatal but not postpartum depressive symptoms. Conversely covariates that show up in Table 5 but not Table 4 are expected to have positive coefficients in the difference analysis, since these covariates are associated with postpartum but not antenatal depressive symptoms. This was confirmed in our analysis: two differentiating factors between antenatal and postpartum scores were a ‘*past history of anxiety’* or a ‘*social status of student/unemployed’*. In the current analysis they were both associated with a drop in the EPDS score by over 2 points from before to after delivery. The reverse was also observed, and a ‘*past history of depression’*, which was significant for postpartum EPDS score only (Table 6) contributed to an increase in the EPDS score by about 2 points.

#### Average of postpartum and antenatal EPDS scores

Significant covariates, which contribute to the average of postpartum and antenatal EPDS scores indicate subject characteristics underlying both scores and are shown in Table 7 (the full model is listed in Suppl. Table 8). As expected, a ‘*past history of PPD*’ as well as ‘*past history of depression’* and ‘a *family history of PPD’* contribute to a higher average EPDS score. In contrast, a ‘*longer gestation’* is associated with a reduction in score. Slightly surprising, obesity might contribute marginally to a reduction in score, but the overall effect was weak. These findings are presented in Fig. 3 that depicts examples of representative case studies.

**Table 7:** Significant covariates of a linear model for average antenatal and postpartum (APA) EPDS scores.

Covariate	Coefficient	p-value
Social status - routine/semi-routine/HW	0.890	0.027
BMI ≥30 - yes	−0.198	0.023
Alcohol pre-pregnancy - yes	0.764	0.039
Past history of PPD - yes	1.737	0.021
Past history depression - yes	2.027	0.001
Family history of PPD - 1st degree relative	1.574	0.002
Gestation length/days	−0.029	0.029

The full model is provided in Suppl. Table 7.

### Prediction of EPDS scores

Finally, we explored the possibility of a stratification of patients based on the results of psychosocial covariates alone without taking EPDS scores into account. To explore whether the regression model is powerful enough for prediction of EPDS scores as well (and not only for finding important covariates of EPDS scores), we compared predictions derived from the four linear models (for AN and PP EPDS, average and difference EPDS) with predictions from a state-of-the-art machine learning prediction algorithm, extreme gradient boosting (xgboost). Details about this approach can be found in the supplementary material. Table 8 shows the result of a ten-fold cross validation (repeated 10 times) of the correlation of predicted scores with the measured scores. Interestingly, the linear models perform comparably (or even slightly better) than the state-of-the-art machine learning prediction algorithm, which depends very little on any statistical assumptions. This indicates that a simple linear model is able to capture most of the signal in the data that is suitable for prediction. Prediction accuracy is highest for antenatal EPDS score, lower for the postpartum score, and in between for the APA score. The available covariates are better suited to predict depression before than after birth. It seems particularly difficult to predict the DPA score, i.e. the change in EPDS score from before to after delivery. However, compare the correlation of 0.19 of predicted postpartum EPDS scores with actual ones using only psychosocial covariates with the correlation of 0.50 of predicting them from prenatal EPDS scores. It is obvious that prenatal EPDS scores are still much better predictors of postpartum EPDS scores than generic psychosocial covariates.

**Table 8:** Ten-fold cross validated correlation of prediction of EPDS scores with original scores based on predictors using penalised linear regression (elastic net), linear regression (linear), and extreme gradient boosting (xgboost).

	Elastic net	Linear	Xgboost
AN EPDS	0.25 (0.02)	0.27 (0.02)	0.24 (0.02)
PP EPDS	0.19 (0.02)	0.18 (0.02)	0.16 (0.02)
DPA EPDS	0.13 (0.03)	0.15 (0.03)	0.13 (0.02)
APA EPDS	0.21 (0.02)	0.22 (0.02)	0.22 (0.02)

In brackets the standard deviation based on 20 random iterations. AN = antenatal; PP = postpartum.

Another way to assess prediction accuracy from a more practical perspective is to inspect PPV and NPV when turning predicted scores into predictions of depressive symptoms at an EPDS cut-off ≥10 (or ≥0 for the DPA score). In our analysis we defined the PPV as the percentage of women predicted to have more severe depressive symptoms, who actually exhibit EPDS scores ≥10, while the NPV is the percentage of women predicted to have minimal symptoms who actually have EPDS <10. The result is shown in Table 9 for the linear and the xgboost predictor (due to the penalisation the absolute score value of the elastic net regression is not representative). The comparatively high correlation for antenatal EPDS score of Table 8 translates into a PPV of around 45% and a NPV of about 85% for the linear model and the xgboost predictor. As expected from the weaker correlation, predictive performance for the other scores is less impressive.

**Table 9:** Ten-fold cross validated positive predictive value (PPV) and negative predictive value (NPV) for prediction of depression based on predictors using linear regression (linear), and extreme gradient boosting (xgboost).

PPV/NPV	Linear %	Xgboost %
AN EPDS	43(8)/86(0.1)	47(5)/86(0.1)
PP EPDS	32(7)/81(0.1)	37(6)/81 (0.1)
DPA	61(1)/50(2)	61(1)/48(2)
APA	38(7)/88(0.1)	36(5)/88(0.1)

In brackets the standard deviation based on 20 random iterations. AN = antenatal; PP = postpartum.

## Discussion

Previous studies^21,24^ have shown a clear correlation between antenatal and postpartum EPDS scores. Our AUC analysis (Fig. 1) and correlation analysis (Fig. 2) show that the same relationship can be found in our data set. Going beyond previous studies we set out to analyse the contribution of psycho-socio-demographic covariates to antenatal, postnatal, and perinatal EPDS scores. The limited correlation of 0.50 between antenatal and postnatal EPDS scores and the extreme differences between these two scores for some patients also raised the question whether there are psychosocial factors contributing to this difference as illustrated in Fig. 3. A first analysis focused on EPDS scores dichotomised using a cut-off value (Table 3). A second analysis used the original EPDS scores for increased power (Tables 4–7). Both analyses resulted in qualitatively similar conclusions about the contributions of various psychosocial covariates. Finally, we explored how far psychosocial factors alone could be used in a clinical setting to predict EPDS scores (Tables 8 and 9).

Our statistical analysis used different types of regression and machine learning methods. Initially we dichotomised EPDS scores to define a binary outcome of depressive symptoms in order to obtain estimates of effects and their significance that are more robust to potential misspecifications of the regression models. This approach is also more likely to be relevant for routine healthcare risk assessment approaches designed around the use of cut-offs; this was followed by development of regression models for the raw EPDS scores in order to increase exploratory power and to detect more subtle effects of covariates without the constraint of cut-offs. Finally we evaluated the predictive performance of the regression models in comparison to a state-of-the-art machine learning prediction method. This complementary but distinct statistical analysis identified a similar set of covariates.

By using EPDS to assess within-person patterns of change of depressive symptoms severity at two distinct time points antenatally (T1) and postpartum (T2) and using a single score cut-off (≥10) we identified three subgroups according to onset and duration or remission of symptoms. An antenatal vs postpartum EPDS score correlation of 0.5002 demonstrates that although there is a clear association, there is also a clear deviation of antenatal EPDS score from postpartum EPDS supporting the hypothesis of a multifactorial aetiology^19^.

Our analysis identified a ‘past history of anxiety or depression’ and ‘socioeconomic deprivation’ as some of the most important risk factors for perinatal depression, in agreement with many previous studies^7,25,26^. A past or family history of depression is a key risk factor for PPD, the average EPDS score between antenatal and postpartum periods, as well as for bigger shifts between postpartum and antenatal EPDS scores. In fact, large prospective population-based studies suggest that the risk of PPD is more than 20 times higher for women with a history of depression compared to women without^27^. This may represent the most important factor to consider when developing a screening program. This finding is in agreement with recent sibling-twins studies that estimate the heritability of PND around 44–54%^28^. Interestingly, one-third of the genetic contribution appears to be unique to PND and not shared with non-perinatal depression, suggesting only partially overlapping genetic etiologies for perinatal and general nonperinatal depression.

**Figure 4:**
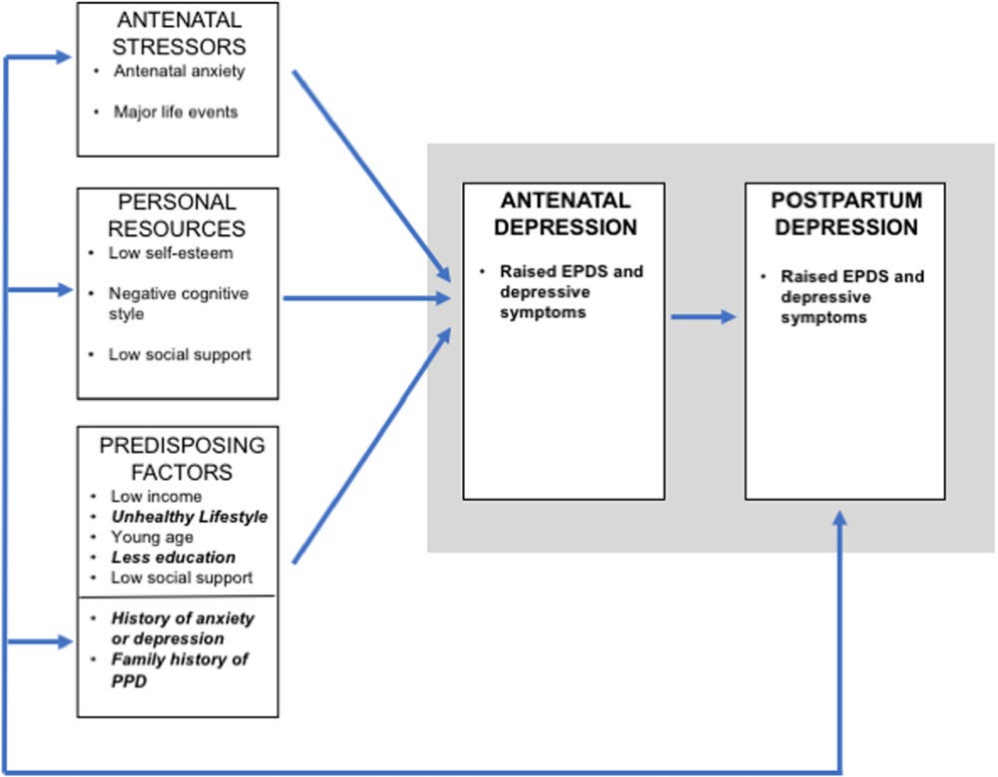
A modified version of the Leigh and Milgrom^8^ biopsychosocial model of perinatal depression, incorporating findings from our study.

Overall, these findings fit well with the conceptual framework describing stress vulnerability and depression in women^5^. Previous history of relevant illness might contribute to individual chronic and acute burdens and accumulation of life stressors that prevent effective adjustment of regulatory mechanisms. Previous studies that employed structural equation modelling to integrate variables such as stressful life events, social support, personality traits, anxiety, coping strategies with postpartum depressive symptoms in a conceptual model of vulnerability to PPD, suggest that women with specific personality traits are more sensitive to the depressogenic effects of adversity and stress events^29^. Similarly, socioeconomic deprivation is another factor clearly linked to stress vulnerability, as observed in low-income populations that exhibit higher levels of stress and impaired coping and depression. Therefore, our findings support and expand the biopsychosocial model of perinatal depression as proposed by Leigh and Milgrom^8^; a modified version of the model incorporating our findings is presented in Fig. 4.

The complex interplay of stress vulnerability factors, precipitating factors associated with adjustment in pregnancy and antenatal stressors, personal resources and coping behaviours and predisposing factors result in a heterogeneous presentation of perinatal depression with affected women experience distinct patterns of onset of symptoms, severity, duration and recurrence. In general, as Tables 4 and 8 show, *antenatal* EPDS scores are better explained by socioeconomic and lifestyle factors (correlation 0.27). These covariates reflect a general level of worry and anxiety, lack of support, harmful lifestyle habits such as excessive alcohol consumption and smoking coupled with lower social status and young age of the pregnant mother.

The protective effect of a higher social status demonstrated in this study points to the benefits of prioritising the most disadvantaged women. Some of these covariates also predict that depressive symptoms might ease after delivery (negative coefficients in Table 6). On the other hand, *postpartum* depressive symptoms are less reliably predicted by the available covariates (correlation 0.18) and are mostly associated with a previous history or a family history of depression and PPD (Table 5). A similarly reduced predictive power of demographic and psychosocial risk factors for PPD compared to AND has been previously reported^5^. These findings suggest a diminished contribution from environmental factors and hint at additional biological-genetic components for PPD.

As Table 9 shows, covariates that are routinely collected from women during antenatal visits, can be used to provide a probability whether an antenatal or postpartum EPDS score ≥10 might be attained by the individual, and are therefore predictive of perinatal depression to some degree. However, the predictive values are still inadequate and identification of additional useful predictors is required (e.g childbirth experience and health of the newborn). As previously mentioned^30^, the exploration of a genetic component, as suggested by the strong contribution of covariates indicating a family history of perinatal depression (Tables 5 and 7), might lead to an improvement in prediction.

Previous studies^21^ suggested that despite acceptable overall discriminatory power, the EPDS has limited predictive accuracy for absolute PPD risk stratification, regardless of the cut-off value used or the trimester of administration. This appears to be related to the presence of multiple risk factors that are postpartum-specific and therefore cannot be included in antenatal screening instruments^31^. To address this, one approach could combine screening for specific psychosocial risk factors alongside EPDS administration. For example, addition of prior history of depression and low partner support to antenatal EPDS seems to yield a more accurate overall prediction of PPD^32^.

### Limitations

A number of study limitations should be considered in the interpretation of these results. Care should be taken when comparing to other populations, since a high majority of our study population were White British and women were generally well educated and identified as ‘supported’. Depressive symptomatology was assessed through the use of a self-report screening instrument rather than clinical assessment, although the EPDS has been reported to have high sensitivity in a large number of studies both in the antenatal period and postpartum^33^. A number of the variables used were binary (yes/no), limiting the conclusions we are able to make. Moreover, the interpretation of personal and family history of depression predicting PPD should be made with caution given that other important factors that contribute to the disease burden related to the childbirth experience^34^ and health of the newborn were not included in the analysis.

Finally, although our study design assessed depressive symptoms at two key time points during pregnancy and postpartum, we recognise that we did not capture the full temporal spectrum of symptoms onset or duration; for example women who experience transient symptoms during the first trimester or beyond 3 months postpartum since there was no follow-up post- 6–10 weeks postpartum, and therefore onset of depressive symptoms after the study period will not have been included in the analysis. The lack of multiple sampling points also did not allow application of modelling tools for identifying multiple un-observed sub-populations, describing longitudinal change within each sub-population, and examining differences in change among sub-populations. Such growth models typically require at least three repeated measures per individual^35^. This will be addressed in future studies.

### Conclusions

This study provides novel evidence for distinct profiles of psycho-socio-demographic characteristics associated with within-person heterogeneity of perinatal depressive symptoms severity, timing of onset and remission. Our results identified key predisposing factors that affect individual’s chronic/acute burden and stress vulnerability and influence depressive risk trajectories during pregnancy and postpartum. This suggests that as women move from the antenatal to the postpartum period, sociodemographic and lifestyle risk factors appear to play a smaller role in risk, and a personal and family history of depression and PPD become increasingly important. Inclusion of covariates, shown to be most predictive of perinatal depression as demonstrated in this study, in a predictive screening system could prioritise resources such as specialist assessment for women most at-risk. It should be emphasised that the covariates and models presented in this study only capture a small, albeit significant, amount of the variability observed in EPDS scores. Further studies, particularly including biological and genetic markers are needed in order to develop an effective screening strategy.

## Methods

### Recruitment, Study Design and Data Collection

Pregnant women were prospectively recruited during routine antenatal visits at the South Warwickshire Foundation NHS Trust, as part of the NIHR CRN Portfolio Study ‘Biomarkers of Perinatal depression. Ethical approval for this study was obtained from the National Research Ethics Service, UK Health Research Authority. All research was performed in accordance with relevant guidelines/regulations and informed consent was obtained from all participants.

Participants were asked to complete the EPDS, a 10 item self-report questionnaire. A detailed description of the study protocol is presented in the Supplementary methods. The antenatal EPDS assessment was carried out between 24–29 weeks gestation (T1) at a hospital visit. All participants were contacted following delivery of their baby and asked to complete a second EPDS at 6–10 weeks postpartum (T2) via post or telephone. Since there is no concensus agreement on the most appropriate assessment time, T1 was chosen to study depressive symptoms during the transition period between 2^nd^ and 3^rd^ trimester where women experience most uncomfortable physical symptoms, such as tiredness, difficulty eating or sleeping and also associated with most dramatic changes in hormonal milieu, especially placental hormones that control stress responses and may trigger rapid mood changes. This is also the period where most women have routine antenatal assessments, therefore translation of this research protocol in clinical practice will not impact on resources allocation in the current healthcare model. Most cases of PPD arise in the 1 to 6 months following childbirth. Generally, it is most common for postpartum depression begin sometime within the first 3 months postpartum therefore T2 was designed to capture the majority of the women who will go to develop PPD^36,37^.

We used a score ≥10 as ‘screening positive’ for both minor and major depressive symptoms (‘high risk’ group). In addition, in some analyses we have interrogated groups with a higher cut-off score ≥13, which is recommended to screen for MDD.

The EPDS was used here in a pre- and post-measures design. For the first stage of the analysis the cut-off score was used to classify patients as high vs low risk for depression during pregnancy or postpartum. This also allowed us to characterise distribution of scores and subgroup patients according to temporal patterns and possible recurrence of depressive symptomatology. For the next stage of the analysis, the EPDS was considered as a continuous scale in order not to constrain the EPDS data to dichotomous groups – this enabled analysis of the relationship between the data collected from the questionnaire variables and EPDS score. Rather than splitting women into high vs low risk groups, for this part of the analysis we instead interrogated the data to investigate which factors contribute higher scores on the EPDS.

Data was collected from the Health, Social and Family Health and Lifestyle Update forms completed at routine antenatal visits as part of normal procedures. A detailed description of the extracted variables is presented in the Supplementary Methods and are displayed in Suppl. Table 1. All questions from the Health, Social and Family forms and Lifestyle Updates are shown in Suppl. Table 1.

This study presents paired observational data from participants that completed the protocol resulting in 960 complete observations (n = 480).

### Statistical analysis

The aim of statistical analysis had three parts: (i) the investigation of distinct patterns of onset and persistence of PND symptoms from antenatal to the postnatal period; (ii) an exploration of the potential for covariate data gathered routinely from women during hospital visits to predict EPDS scores: (iii) to test the degree to which severity of antenatal and postpartum depression as well as the change in severity across the two periods was associated with distinct covariates risk profiles. Details of the statistical analysis approach is presented in the Supplementary Methods.

### Ethics approval

The study protocol was approved by the NIHR Clinical Research Network as a Portfolio study (Study IRAS ID: 21234). The current study protocol REC reference is 09/H1203/69 and was approved on the 14-July 2014.

## Electronic supplementary material

Supplementary methods and data

## Data Availability

The datasets analysed during the current study are available from the corresponding author on reasonable request.
