# Supplementary material for: Modelling of psychosocial and lifestyle predictors of peripartum depressive symptoms associated with distinct risk trajectories: a prospective cohort study
Source: Sci Rep. 2018 Aug 24;8:12799. doi: 10.1038/s41598-018-30874-z (PMC6109131; doi:10.1038/s41598-018-30874-z)
Supplement: Supplementary file 1 — Supplementary methods and data [file 41598_2018_30874_MOESM1_ESM.docx]

## Modelling of psychosocial and lifestyle predictors of peripartum depressive symptoms associated with distinct risk trajectories: a prospective cohort study

## S. English^1^, A. Steele^2^, A. Williams^3^, J. Blacklay^3^, O. Sorinola^1,3^, L. Wernisch^2^, D.K Grammatopoulos^1,4^

##

## ^1^Translational Medicine, Warwick Medical School, Coventry, UK

## ^2^MRC Biostatistics Unit, Institute of Public Health, Cambridge, UK

## ^3^ South Warwickshire Hospital Foundation Trust, Warwick, UK

## ^4^ Institute of Precision Diagnostics and Translational Medicine, Dept of Pathology, University Hospital Coventry and Warwickshire, Coventry, UK

##

**SUPPLEMENTARY MATERIAL**

**SUPPLEMENTARY METHODS**

**Recruitment, Study Design and Data Collection:** Participants were asked to complete the EPDS, a 10 item self-report questionnaire. The EPDS is validated for antenatal use, as a pre-screening tool for depression and in particular in research. It does not include questions about somatic complaints, fatigue and changes in appetite, as these complaints would not help to distinguish depressed from non-depressed women^1^. The antenatal EPDS assessment was carried out between 24-29 weeks gestation at a hospital visit. All participants were contacted following delivery of their baby and asked to complete a second EPDS at 6-10 weeks postpartum via post or telephone. The timing of the follow-up EPDS was based on previous studies to include the peak incidence periods for PND^2-4^.

The EPDS is scored on a scale from 0 to 30. We used a score ≥10 as ‘screening positive’ for both minor and major depressive symptoms (‘high risk’ group) as recommended by the American Academy of Paediatrics. This cut-off score has a wide-ranging reported sensitivity for detecting MDD, from 67-100% and for major or minor depression ranges from 63-84%^5^.

Specificity was similar for detecting MDD and detecting both major and minor depression and is ranged from 71-90%. In addition, in some analyses we have interrogated groups with a higher cut-off score ≥13, which is recommended to screen for MDD with sensitivity that range from 67-95% and specificity 90-99% (in the Systematic Evidence Review for the US Preventive Services Task Force^5^).

Patients were involved during the preliminary design of the study especially regarding the methods of remote communication post-delivery.

Data was further extracted from the Health, Social and Family Health forms, which are routinely completed during the initial antenatal booking visit by a midwife. This is used routinely across the NHS using standardised questionnaires, and is designed to obtain information about pre-existing medical conditions and socio-demographic characteristics, in addition to questions about pregnancy and lifestyle. Lifestyle Update forms were also completed at routine antenatal visits as part of normal procedures, from which data was also collected for this study. These forms include a number of known risk factors for PND and these measures were included in the present analysis.

All variables from these forms for which data was extracted for this study are displayed in Suppl Table . For example, ‘*anxiety’* is a significant risk factor for antenatal depressive symptoms. This link has been well established and is likely attributable to the pressures of becoming a young mother including the potential financial strain^6 7^. Covariate `*Social status*' is indicative of socioeconomic status (SES), which is a well-established risk factor for postpartum depression^8 9^. This study used occupational status as an indicator of social status, and therefore more options for returning to work after having children could be beneficial. Low social status also often indicates adversity in life.

All questions from the Health, Social and Family forms and Lifestyle Updates are shown in Suppl Table 1. From the questionnaire 21 covariates were extracted, 4 of them numerical integer values (with ranges indicated in Suppl Table 1), 17 factorial covariates with factor levels as described in Suppl Table 1, which also shows the number of individuals per factor level. Initial analysis showed that the majority of the women in the data set were White British (84.6%), educated to a minimum of degree level (55.9%), were in the 30-34 age group (37.7%), had a 1st trimester BMI of 18.5 – 24 (47.9%), had a social status of higher managerial/professional (35.2%), were not on any medication (84.6%) or Omega 3 supplements (76.3%), had consumed alcohol before pregnancy (68.3%) but did not drink during pregnancy (97.3%). The majority did not have a history of anxiety (86.7%), or depression (89.6%), did not smoke at any time prior to pregnancy (88.8%), did not smoke during pregnancy (96.5%) and had no family history of PPD (84.0%). The great majority were supported (98.3%), 44.8% had one child prior to the pregnancy, and 52.1% delivered a baby boy. All variables and frequencies are displayed in Suppl Table 1. The hard copies of EPDS questionnaires were collected, both at the antenatal and postpartum time points, resulting in matched paired observational data.

**Statistical analysis:**

**Multinomial logistic regression:** A number of potential risk factors for PND, which were identified in the published literature and available in this study, were selected for in the present analysis such as age, ethnicity and social status among many others (Suppl Table 1). All selected variables were first analysed for statistical significance of associations between potential risk factors and pre/postnatal depression through chi-squared (χ2) tests. Odds ratios (OR) and the associated 95% confidence interval (CI) were obtained for variables that demonstrated statistical significance during chi-squared analysis (Suppl Tables 2&3). Pre- and post-delivery variables for EPDS scores were created to conduct independent analysis between the risk factors.

A p-value of <0.05 was considered indicative of a statistically significant difference. All risk factors with a p<0.05 in univariable analysis, either pre-delivery or post-delivery, were identified and included in a multinomial logistic regression model to study the relationship between group classification and risk factors with STATA commands *mlogit* and *mlogit,rrr* to obtain relative risk and p-values. To simplify analysis four individuals with missing data were excluded resulting in N=476. Covariates with low numbers in some levels were merged as shown in Suppl Table 1. Overall low risk group (Group 0) was used as the baseline category. Relative risk values were calculated to provide a better understanding of the magnitude of association between risk factors and each group. Statistical analyses were conducted with IBM SPSS Statistics Version 24 and STATA/SE 14.0.

**Penalised regression and prediction:** After the first phase of the analysis using logistic regression, which identified covariates associated with EPDS categories, we aimed for a more detailed view of factors contributing to PND and particularly to the difference between antenatal and postpartum EPDS scores. However, including all available covariates, of which many had a multitude of levels, including pairwise interactions, requires some form of systematic variable selection, since the number of variables easily exceeds the sample size. Penalised regression is a widely used form of regression for models with a large number of covariates. Therefore, in order to find the best combination of predictors we applied a linear model with elastic net regularization (for details see Zou and Hastie, 2005^10^) as implemented in the R function *glmnet* from the glmnet package. We used default settings with the parameter alpha set to 0.5, that is, the Lasso and ridge regularisation were given equal weight. This type of regularisation encourages a sparse regression where unimportant covariates are removed, while at the same time keeping highly correlated variables in the model to facilitate interpretation of the model, when two or more correlated covariates explain the outcome equally well. Moreover, the regression coefficients are shrunk towards zero, stabilising the estimation of coefficients and preventing large positive and negative coefficients compensating each other in the case of correlated covariates. The regularisation strength parameter *lambda* required by the *glmnet* procedure was selected by cross-validation as implemented in the R function *cv.glmne*t with default parameters.

Since there is no universally accepted method for assigning *p*-values to coefficients obtained by sparse regression, and these coefficients also show considerable bias, we fitted a standard linear model using the covariates selected in the penalised regression to obtain unbiased estimates of the coefficients as well as corresponding *p*-values. Note that p-values obtained in this way have to be interpreted with caution, since the data have been used twice: first to find a model resulting in a data dependent set of hypotheses on regression coefficients, and then second to test these data dependent hypotheses. However, as has been argued recently [S Zhao, A Shojaie, D Witten, In Defense of the Indefensible: A Very Naive Approach to High-Dimensional Inference, <https://arxiv.org/abs/1705.05543>] using lasso regression for model selection might not invalidate interpretation of p-values of a subsequent regression analysis, since under many circumstances the lasso selected covariates behave as if they were selected deterministically, at least asymptotically. Our data are also not in this sense very high dimensional. Moreover, our aim was an exploratory analysis which will need formal confirmation in follow up studies.

One of the aims of the current analysis is to explore the possibility of a stratification of patients based on the results of the questionnaire. To explore whether the regression model would be suitable for prediction as well (and not only to find important covariates of EPDS scores), we compared predictions derived from the model with predictions from a state-of-the-art machine learning prediction algorithm, extreme gradient boosting, in the form of function *xgboost* from the xgboost R package. The following hyperparameters required by this function were found by optimising a ten-fold cross validated prediction error: booster="gblinear", max_depth = 1, eta = 1, nrounds = 3, objective = "reg:linear".

To simplify the regression analysis for the regression 6 individuals of the 480 with missing values in some covariates were removed. Since this amounts to about 1% of the data, the effect on the statistical results were considered negligible.

**SUPPLEMENTARY RESULTS**

**EPDS score distributions**

In our study population we identified a prevalence of 11.5% for antenatal (n=55) and 14.8% (n=71) for postpartum depressive symptoms. This falls within the estimated 10-15% shown by other studies^11-13^. Analysis of the risk trajectory defined by EPDS scores during pregnancy and postpartum period identified four groups of women. Those with raised EPDS scores postpartum only (group 1) represented the largest group of ‘high risk’ patients with 45% of the total (n=101). Group 1 had a median antenatal EPDS score of 6 and median postpartum score of 12. The proportion of women with raised EPDS either antenatally (group 2) or at both assessment time points (group 3) was 30% and 25%, respectively. Group 2 has a median antenatal-postpartum EPDS scores of 13 and 6, whereas Group 3 exhibited the highest median values with an antenatal EPDS median score of 15 and postpartum EPDS of 16. Patients from group 3 (high risk both antenatally and postpartum) appear to exhibit the most severe symptoms according to EPDS scores; median values of both antenatal and postpartum scores were within the moderate depression range as proposed by McCabe-Beane^14^. In contrast median values of raised EPDS in groups 1, and 2 were within the mild depression range.

At a cut-off of 10, the screening identified a prevalence of 11.5% for antenatal (n=55) and 14.8% (n=71) for postpartum depressive symptoms. Antenatal EPDS scores ranged from 0-25 and postpartum EPDS scores ranged from 0-22 with mean scores of 5.42 and 5.78 respectively. It has been proposed that the EPDS score correlates with symptoms severity: scores of 0-9 are suggestive of minimal depression, 10-12 of minor depression and ≥13 of major depression. In our cohort of the 55 women screening positive for antenatal depressive symptoms, 45% had scores in the 10-12 range (at risk of minor depression) and 54% had scores ≥13 (at risk of major depression). Of the 71 women screening positive for postpartum depressive symptoms, 48% had EPDS scores in the 10-12 range, whilst 52% scored ≥13.

Using a cut-off of 10, the distribution of ‘at-risk’ patients across the three groups was 45% in group 1, 37% in group 2 and 28% in group 3. Increasing the cut-off to 13 (to increase specificity of EPDS and include patients with MDD symptoms only) did not significantly alter the relative distribution of each group, with a similar distribution of 43%, 30% and 27% for groups 1, 2 and 3 respectively (*Supplementary* *Figure* 1b). However, decreasing the cut-off to 8 shifted the relative distribution of ‘at-risk’ patients and resulted in approximately equal numbers across the three groups (34% in group 1, 32% in group 2 and 34% in group 3): this redistribution was characterised mainly by a increase in the size of both groups 2 and 3 and a concomitant reduction by 10% in the size of group 1. *Supplementary Figure* *1a* shows that our cohort contained a larger number of women scoring 8/9 in the antenatal EPDS compared to postpartum EPDS, resulting in the inclusion of more women at risk of antenatal depression in groups 2 and 3 when the cut-off was lowered to 8.

**Shifts between severity risk categories**

Supplementary Table 2 sub-classified women who screened ‘positive’ in the EPDS into two risk categories: risk of minor depression (10-12), and major depression (≥13). In addition to moving from a negative to a positive screen for depressive symptoms and vice versa, women also shifted in severity category. These severity shifts were also bidirectional with both increases as well as decreases in EPDS scores from antenatal to postpartum period were observed. In detail, 79% (n=379) of women remained in the low risk category throughout the period of the study (24w of pregnancy and up to 10w postpartum), whereas 21% of women had raised risk of perinatal depression during pregnancy or postpartum or both. Group 1 included 46 women who were low risk during pregnancy, but moved to high risk postpartum: 64% exhibited EPDS scores in the minor depressive risk category (10-12) and 37% had scores in the MDD risk category (≥13). Likewise, group 2 contained women (n=30) who shifted to low-risk postpartum (<10) from high risk during pregnancy: 60% had antenatal EPDS scores in the minor category, whereas 40% had scores in the MDD category. Group 3 included women (n=4) who had their antenatal EPDS scores increased from the minor risk category to MDD risk category postpartum, whilst only two women high (≥13) antenatal EPDS scores were lowered to the minor category postpartum. Moreover, in 64% women in group 3, no differences in the severity category were observed.

**Bivariate chi-squared analysis**

For antenatally raised EPDS significantly associated factors were social status, history of depression, anxiety, age, support, education, underlying illness associated medication, ethnicity and gestational length (Suppl Table 3). For postpartum raised EPDS significantly associated factors were history of PPD, history of depression, and family history of PPD (Suppl Table 4).

**REFERENCES**

1. Murray D, Cox, J. Screening for depression during pregnancy with the edinburgh depression scale (EDDS). *Journal of Reproductive and Infant Psychology* 1990;8(2):99-107.

2. Dennis CL, Hodnett E. Psychosocial and psychological interventions for treating postpartum depression. *Cochrane Database Syst Rev* 2007(4):39.

3. Verkerk GJM, Pop VJM, Van Son MJM, et al. Prediction of depression in the postpartum period: a longitudinal follow-up study in high-risk and low-risk women. *Journal of Affective Disorders* 2003;77(2):159-66.

4. Cox JL, Murray D, Chapman G. A CONTROLLED-STUDY OF THE ONSET, DURATION AND PREVALENCE OF POSTNATAL DEPRESSION. *British Journal of Psychiatry* 1993;163:27-31.

5. O'Connor E, Rossom RC, Henninger M, et al. Primary Care Screening for and Treatment of Depression in Pregnant and Postpartum Women Evidence Report and Systematic Review for the US Preventive Services Task Force. *Jama-Journal of the American Medical Association* 2016;315(4):388-406. doi: 10.1001/jama.2015.18948

6. Bodecs T, Szilagyi E, Cholnoky P, et al. PREVALENCE AND PSYCHOSOCIAL BACKGROUND OF ANXIETY AND DEPRESSION EMERGING DURING THE FIRST TRIMESTER OF PREGNANCY: DATA FROM A HUNGARIAN POPULATION-BASED SAMPLE. *Psychiatr Danub* 2013;25(4):352-58.

7. Rich-Edwards JW, Kleinman K, Abrams A, et al. Sociodemographic predictors of antenatal and postpartum depressive symptoms among women in a medical group practice. *J Epidemiol Community Health* 2006;60(3):221-27. doi: 10.1136/jech.2005.039370

8. Robertson E, Grace S, Wallington T, et al. Antenatal risk factors for postpartum depression: a synthesis of recent literature. *General Hospital Psychiatry* 2004;26(4):289-95. doi: 10.1016/j.genhosppsych.2004.02.006

9. Beck CT. Predictors of postpartum depression - An update. *Nursing Research* 2001;50(5):275-85. doi: 10.1097/00006199-200109000-00004

10. Zou H, Hastie T. Regularization and variable selection via the elastic net. *J R Stat Soc Ser B-Stat Methodol* 2005;67:301-20. doi: 10.1111/j.1467-9868.2005.00503.x

11. Ohara MW, Swain AM. Rates and risk of postpartum depression - A meta-analysis. *International Review of Psychiatry* 1996;8(1):37-54. doi: 10.3109/09540269609037816

12. Teixeira C, Figueiredo B, Conde A, et al. Anxiety and depression during pregnancy in women and men. *Journal of Affective Disorders* 2009;119(1-3):142-48. doi: 10.1016/j.jad.2009.03.005

13. Nonacs R, Cohen LS. Postpartum mood disorders: Diagnosis and treatment guidelines. *Journal of Clinical Psychiatry* 1998;59:34-40.

14. McCabe-Beane JE, Segre LS, Perkhounkova Y, et al. The identification of severity ranges for the Edinburgh Postnatal Depression Scale. *Journal of Reproductive and Infant Psychology* 2016;34(3):293-303. doi: 10.1080/02646838.2016.1141346

**SUPPLEMENTARY TABLES**

**Supplementary table 1***:* Questions asked in the Health, Social and Family forms with levels of factors and number of individuals. The first level of each covariate serves as base level in the linear model.

| **Covariates** | **Question** | **Levels** | **N (%)** |
| --- | --- | --- | --- |
| **Education** | To what level have you been in education? | Did not take any exams | 0 (0.0) |
|  |  | > 16 years | 52 (10.8) |
|  |  | > 18 years | 59 (12.3) |
|  |  | Diploma Level | 101 (21.0) |
|  |  | Degree Level | 166 (34.6) |
|  |  | Professional | 43 (9.0) |
|  |  | Post Graduate | 49 (10.2) |
|  |  | Doctorate | 10 (2.1) |
| **Education groups** | To what level have you been in education? | Post 16  Diploma  1st Degree  Higher Degree | 111 (23.3)  101 (21.2)  164 (34.5)  100 (21.0) |
| **Ethnicity** | What is your ethnicity? | White British | 406 (84.6) |
|  |  | Indian | 20 (4.2) |
|  |  | Asian | 11 (2.3) |
|  |  | Other white | 27 (5.6) |
|  |  | Black Caribbean | 1 (0.2) |
|  |  | Black African | 0 (0.0) |
|  |  | Mixed | 11 (2.3) |
|  |  | Other | 4 (0.8) |
| **Ethnicity groups** | What is your ethnicity? | White British  Indian  Asian  Remaining minorities | 403 (84.7)  19 (4.0)  11 (2.3)  43 (9.0) |
| **Social Status** | What is your current employment status/level? | Higher managerial/professional | 169 (35.2) |
|  |  | Diploma level professional | 82 (17.1) |
|  |  | Self employed | 31 (6.5) |
|  |  | Low level supervisory/technical | 37 (7.7) |
|  |  | Semi routine/routine/HW | 147 (30.6) |
|  |  | Unemployed/student | 14 (2.9) |
| **Social groups** | What is your current employment status/level? | Higher managerial/professional  Diploma level professional  Self employed  Low level supervisory/technical  Unemployed/routine work | 166 (34.9)  82 (17.2)  31 (6.5)  37 (7.8)  160 (33.6) |
| **Alcohol (pre-pregnancy)** | Did you drink alcohol during the 12 months before conception? | No  Yes | 152 (31.7)  328 (68.3) |
| **Drinking (in pregnancy)** | Do you drink alcohol during pregnancy? | No  Yes | 467 (97.3)  13 (2.7) |
| **Smoking (pre-pregnancy)** | Were you a smoker during the 12 months before conception? | No  Yes | 426 (88.8)  54 (11.3) |
| **Cigarettes (in pregnancy)** | Do you currently smoke? | No | 463 (96.5) |
|  |  | Yes | 17 (3.5) |
| **Supported** | Do you feel supported? | No | 8 (1.7) |
|  |  | Yes | 472 (98.3) |
| **Parity (number of previous births)** | Number of previous births? | 0 | 202 (42.1) |
|  |  | 1 | 215 (44.8) |
|  |  | 2 | 41 (8.5) |
|  |  | 3 | 13 (2.7) |
|  |  | 4 | 6 (1.3) |
|  |  | 5 | 2 (0.4) |
|  |  | 6 | 1 (0.2) |
| **Age** | Maternal age at 12w? | <18 | 1 (0.2) |
|  |  | 18-24 | 44 (9.2) |
|  |  | 25-29 | 101 (21.0) |
|  |  | 30-34 | 181 (37.7) |
|  |  | 35-39 | 114 (23.8) |
|  |  | 40-45 | 37 (7.7) |
|  |  | >45 | 2 (0.4) |
| **Age groups** | Maternal age at 12w? | <25  25-29  30-34  35-39  40+ | 45 (9.5)  101 (21.2)  178 (37.4)  114 (24.0)  45 (9.5) |
| **BMI** | Body Mass Index (BMI) at 12w? | < 18.5 | 17 (3.5) |
|  |  | 18.5 - 24 | 230 (47.9) |
|  |  | 25-29 | 103 (21.5) |
|  |  | 30-34 | 91 (19.0) |
|  |  | 35-39 | 23 (4.8) |
|  |  | 40-44 | 12 (2.5) |
|  |  | > 45 | 4 (0.8) |
| **BMI ≥30** | BMI ≥30 at 12w | No  Yes | 350 (72.9)  130 (27.1) |
| **Co-morbidities and Medication** | Do you currently take any medication? | No | 406 (84.6) |
|  |  | Thyroxine | 20 (4.2) |
|  |  | Anticoagulant | 22 (4.6) |
|  |  | Asthma drugs | 10 (2.1) |
|  |  | Analgesia | 3 (0.6) |
|  |  | Other | 19 (4.0) |
| **Medication groups** | Do you currently take any medication? | No  Yes | 402 (84.5)  74 (15.6) |
| **Omega 3** | Do you currently take an omega-3 supplement? | No | 366 (76.3) |
|  |  | Yes | 114 (23.8) |
| **Anxiety** | Have you ever had anxiety? | No | 416 (86.7) |
|  |  | Yes | 64 (13.3) |
| **Past History of Depression** | Have you ever had depression? | No |  |
|  |  | Yes | 430 (89.6) |
|  |  |  | 50 (10.4) |
| **Sex** | Sex of baby? (completed after delivery) | Girl | 227 (47.9) |
|  |  | Boy | 247 (52.1) |
| **Family History PPD** | Has anyone in your family ever had postnatal depression? | No | 403 (84.0) |
|  |  | 1st degree relative | 68 (14.2) |
|  |  | Other family | 9 (1.9) |
| **Past History PPD** | Have you ever had postnatal depression? | No | 453 (94.4) |
|  |  | Yes | 27 (5.6) |
| **Gestation (days)** | Total gestational length (completed after delivery) | Mean of study population | 277.14 |
| **Weight of baby (grams)** | Weight of baby at birth (completed after delivery) | Mean of study population | 3502.67 |

**Supplementary table 2:** Cross-tabulation of antenatal (T1) and postpartum (T2) depressive symptoms risk according to EPDS risk categories 0-9 (minimal depression), 10-12 (minor depression) and ≥13 (major depression).

|  | | **T2 EPDS cut-off** | | | Total |
| --- | --- | --- | --- | --- | --- |
|  |  | *≤ 9* | *10-12* | *≥13* |  |
| **T1 EPDS**  **cut-off** | *≤ 9* | 379 | 29 | 17 | 425 |
|  | *10-12* | 18 | 3 | 4 | 25 |
|  | *≥13* | 12 | 2 | 16 | 30 |
| Total | | 409 | 34 | 37 | 480 |

**Supplementary Table 3:** Odds ratios and 95% CI for all variables which demonstrated significance in Chi-squared analysis for antenatal depressive symptoms.

| Factor | OR | 95% CI |
| --- | --- | --- |
| **Age** | 1.064421 | 0.7882357, 1.437377 |
| **Social status** | 1.465595 | 1.191677, 1.802476 |
| **Support** | 0.4204886 | 0.0773037, 2.287221 |
| **Education** | 0.7729944 | 0.6347195, 0.9413929 |
| **Medication** | 1.034493 | 0.8022022, 1.334048 |
| **History of depression** | 2.295905 | 1.073715, 4.909291 |
| **History of anxiety** | 2.089739 | 1.025862, 4.256915 |
| **Gestational length** | 0.9734467 | 0.955314, 0.9919235 |
| **Ethnicity** | 1.207497 | 1.00187, 1.455328 |

**Supplementary Table 4:** Odds ratios and 95% CI for all variables which demonstrated significance in Chi-squared analysis for postpartum depressive symptoms.

| **Factor** | **OR** | **95% CI** |
| --- | --- | --- |
| **History of PND** | 3.287746 | 1.287778, 8.393746 |
| **Family history of PND** | 1.530038 | 0.8929046, 2.621797 |
| **History of depression** | 3.208556 | 1.661414, 6.196427 |

**Supplementary Table 5:** All covariates which contribute to antenatal EPDS score in elastic net regression analysis

| **Covariate** | **Elastic net** | **Coefficient** | **p-value** |  |
| --- | --- | --- | --- | --- |
| (Intercept) | 10.278 | 12.864 | 0.003 | * |
| **Age18-24** | 0.926 | 1.154 | 0.109 |  |
| **Age 25-29** | -0.042 | -0.491 | 0.315 |  |
| **Age >45** | 3.262 | 6.225 | 0.144 |  |
| **Ethnicity - Black Caribbean** | -2.079 | -5.819 | 0.159 |  |
| **Ethnicity - Mixed** | 0.787 | 1.782 | 0.164 |  |
| **Ethnicity - Other** | 1.308 | 3.144 | 0.133 |  |
| **Social status - Low level supervisory/technical** | 0.221 | 1.23 | 0.108 |  |
| **Social status - Semi routine/routine/HW** | 1.071 | 1.768 | <0.001 | * |
| **Social status - Unemployed/student** | 2.673 | 3.887 | 0.004 | * |
| **Supported - Yes** | -1.471 | -1.627 | 0.339 |  |
| **Education >18 yrs** | -0.667 | -1.373 | 0.033 | * |
| **Education - Degree** | -0.084 | -0.294 | 0.544 |  |
| **Education - Diploma** | 0.474 | 0.466 | 0.395 |  |
| **Alcohol - Yes** | 0.351 | 0.813 | 0.049 | * |
| **Medication - Anticoagulant** | 0.847 | 1.405 | 0.134 |  |
| **BMI ≥30 - Yes** | -0.581 | -0.927 | 0.037 | * |
| **Omega3 - Yes** | 0.027 | 0.381 | 0.404 |  |
| **Past history of PPD - Yes** | 1.005 | 1.356 | 0.107 |  |
| **Past history of depression - Yes** | 0.732 | 0.834 | 0.21 |  |
| **Family history of PPD, 1st degree relative - Yes** | 0.909 | 1.391 | 0.012 | * |
| **Past history of anxiety - Yes** | 1.874 | 2.314 | <0.001 | * |
| **Gestation / days** | -0.016 | -0.027 | 0.063 |  |
| CV / R2 / R2 adj | 18.569 | 0.204 | 0.165 |  |

**Supplementary Table 6:** All covariates which contribute to postpartum EPDS score in elastic net regression analysis.

| **Covariate** | **Elastic net** | **Coefficient** | **p-value** |  |
| --- | --- | --- | --- | --- |
| (Intercept) | 6.458 | 12.399 | 0.005 | * |
| **Education - Diploma** | 0.262 | 0.961 | 0.057 |  |
| **Medication - Asthma** | 0.528 | 2.648 | 0.065 |  |
| **Past history of PPD - Yes** | 0.998 | 2.204 | 0.014 | * |
| **Past history of depression - Yes** | 1.933 | 2.788 | <0.001 | * |
| **Family history of PPD, 1st degree relative - Yes** | 0.893 | 1.779 | 0.003 | * |
| **Gestation / days** | -0.004 | -0.027 | 0.085 |  |
| CV / R2 / R2 adj | 21.095 | 0.108 | 0.096 |  |

**Supplementary Table 7:** All covariates which contribute to the difference between antenatal and postpartum EPDS scores in elastic net regression analysis

| **Covariate** | **Elastic net** | **Coefficient** | **p-value** |  |
| --- | --- | --- | --- | --- |
| (Intercept) | 0.747 | 1.174 | <0.001 | * |
| **Age 18-24** | -0.784 | -1.1 | 0.14 |  |
| **Ethnicity - Other** | -0.056 | -2.648 | 0.234 |  |
| **Social status - Semi routine/routine/HW** | -0.232 | -0.858 | 0.069 |  |
| **Social status - Unemployed/student** | -1.391 | -2.664 | 0.04 | * |
| **Education - Doctorate** | 0.162 | 1.619 | 0.278 |  |
| **Education - Post graduate** | -0.078 | -0.816 | 0.228 |  |
| **Drinking - Yes** | -0.387 | -1.301 | 0.325 |  |
| **Smoking - Yes** | -0.952 | -1.185 | 0.086 |  |
| **Medication - Anticoagulant** | -0.156 | -1.525 | 0.115 |  |
| **Medication - Asthma drugs** | 0.685 | 2.452 | 0.084 |  |
| **Past history of depression -Yes** | 0.931 | 2.036 | 0.003 | * |
| **Past history of anxiety-Yes** | -1.543 | -2.565 | <0.001 | * |
| CV / R2 / R2 adj | 20.158 | 0.108 | 0.085 |  |

**Supplementary Table 8**: All covariates which contribute to the average of antenatal and postpartum EPDS scores in elastic net regression analysis.

| **Covariate** | **Elastic net** | **Coefficient** | **p-value** |  |
| --- | --- | --- | --- | --- |
| (Intercept) | 9.472 | 13.774 | <0.001 | * |
| **Age 18-24** | 0.067 | 0.682 | 0.275 |  |
| **Social status - Semi routine/routine/HW** | 0.3 | 0.89 | 0.027 | * |
| **Social status - Unemployed/student** | 0.543 | 1.876 | 0.11 |  |
| **Supported - Yes** | -1.184 | -1.626 | 0.289 |  |
| **Education >18 yrs** | -0.053 | -0.791 | 0.138 |  |
| **Education - Diploma** | 0.542 | 0.858 | 0.052 |  |
| **Alcohol - Yes** | 0.09 | 0.764 | 0.039 | * |
| **Medication - Asthma drugs** | 0.137 | 1.439 | 0.228 |  |
| **BMI ≥30 - Yes** | -0.198 | -0.889 | 0.023 | * |
| **Past history of PPD - Yes** | 1.148 | 1.737 | 0.021 | * |
| **Past history of depression - Yes** | 1.514 | 2.027 | 0.001 | * |
| **Family history of PPD, 1st degree relative - Yes** | 0.904 | 1.574 | 0.002 | * |
| **Past history of anxiety - Yes** | 0.458 | 0.755 | 0.145 |  |
| **Gestation / day** | -0.012 | -0.029 | 0.029 | * |
| CV / R2 / R2 adj | 14.953 | 0.165 | 0.14 |  |

**Supplementary Figure 1:** (a) Distribution of antenatal and postpartum EPDS scores within the cohort. Dashed line indicates cut-off score of 10 used to indicate depressive risk. (b) Comparison of distributions using cut-off scores of 8, 10 and 12.
